# The relationship between selected socioeconomic factors and thinness among Polish school-aged children and adolescents

**DOI:** 10.1007/s00431-017-2912-1

**Published:** 2017-04-28

**Authors:** Beata Gurzkowska, Zbigniew Kułaga, Aneta Grajda, Magdalena Góźdź, Małgorzata Wojtyło, Mieczysław Litwin

**Affiliations:** 1 Public Health Department, The Children’s Memorial Health Institute, al. Dzieci Polskich 20, 04-730 Warsaw, Poland; 2 Department of Nephrology and Arterial Hypertension, The Children’s Memorial Health Institute, al. Dzieci Polskich 20, 04-730 Warsaw, Poland

**Keywords:** Thinness, Socioeconomic factors, Birth weight, Height

## Abstract

The analysis was performed on a database including 17,427 records of subjects aged 7–18 years, randomly sampled from a population of Polish students. Thinness was determined using international cut-off points, defined to pass through body mass index of 18.5 kg/m^2^ at the age of 18. The weighted prevalence of thinness and odds ratios with 95% confidence interval were estimated for gender, birth weight, level of schooling and school location, gross domestic product (GDP) per inhabitant, family income and maternal education. Body height was analysed according to body mass and birth weight categories. The prevalence of thinness was higher among children with low birth weight and among girls. The prevalence of thinness decreased with increasing GDP per inhabitant. In analysis by level of schooling: primary-middle-secondary, prevalence of thinness decreased among boys and increased among girls. Thin students were significantly shorter than other students, and thin girls less likely participated in physical education.

*Conclusion*: Gender and socioeconomic factors are important determinants of thinness among Polish children and adolescents. Public health strategies should address family eating practices to prevent negative effects of weight deficit, especially among girls/children from low GDP regions.

**Table:** 

**What is Known:** • *Socioeconomic factors and gender influence weight status of children and adolescents.*
**What is New**: • *The first time the prevalence and determinants of thinness based on data from a nationally representative, weighted sample of Polish children aged 7–18 years were presented.* • *The analysis shows how gender and socioeconomics determinants influence the prevalence of thinness among children and adolescent in post-transformation country and can be used to international comparisons.*

## Introduction

Nutritional status assessment plays an important role in evaluating the health status of the population. Measurements of weight and height and analysis of body mass index (BMI) are a simple method which is used in the nutritional status assessment of children and adolescents [18]. Both thinness and overweight affects children’s development and reaching their optimal potential of good health [22].

Being thin may be due to both genetic and external factors, related to the child’s environment. Inadequate intake of energy and nutrients that may be associated with the limited resources of food, stress or an unbalanced diet used in a medical disorder (e.g. allergy) or poor eating habits are the most common causes of low weight to height ratio in adolescents. Insufficient supply of protein, energy or micronutrients may lead to malnutrition, which may also result in reducing the body’s resistance to pathogens, delayed physical and intellectual development or cause metabolic disorders. Underweight, although to a lesser extent than obesity, affects the morbidity of children and adolescents [15].

The aim of this study was to investigate the prevalence of thinness among children and adolescents in the context of socioeconomic determinants, gender, body height and birth weight.

## Materials and methods

### Study population and sampling

This study used nationally representative data from the OLAF study (“Elaboration of reference blood pressure ranges for children and adolescents in Poland”—PL0080 (OLAF)). The study protocol was approved by the Bioethics Committee of the Children’s Memorial Health Institute (CMHI). The studied population comprised school students, who between school years 2007–2008 and 2009–2010 attended primary, middle and secondary schools. Participants were selected in a two-stage random sampling procedure. The primary sampling unit (PSU) was a school sampled from an all-schools-in-Poland sampling frame provided by the Polish Ministry of Education. The schools were selected by stratified sampling with probability proportional to the size of the unit. In respect of primary and middle schools, the strata were urban/rural, whereas for secondary schools, the kind of school. In the second stage, the participants were randomly selected based on the number of students in a given school. There were 24,814 students randomly selected and invited to take part in the study, of whom 17,573 children and adolescents (8396 boys) participated in this study (response rate 71%).

### Data collection

Informed consent for participation in the study was obtained in writing from the parents (or guardians) of students under the age of 18 years, as well as from participants over the age of 16 years. Body height and body weight were measured according to the procedures delineated in the OLAF study protocol and described previously [13]. Height was measured in duplicate using a stadiometer (SECA 214) in the standing position (without shoes), to the nearest millimetre. Body weight was measured in light underwear twice, using a digital, medical scale (Radwag WPT 100/200), and was recorded to the nearest 0.05 kg. A detailed description of the sampling, as well as description of procedures for anthropometric measurements, is included in earlier publication [13].

BMI was calculated as the ratio of weight in kilograms and the square of height in meters.

The age of the study participant was calculated from the difference between the date of measurement and the date of birth. Thinness was defined according to gender and age-specific BMI cut-off points by Cole TJ et al. [4]; overweight and obesity was defined based on BMI cut-off points according to the International Obesity Task Force (IOTF) [3]. Body weight categories were assigned using LMSgrowth (downloaded from the website http://homepage.mac.com/tjcole/FileSharing1.html).

Data on health status (including birth weight), as well as the socio-economic and environmental situation of the student, were collected with the use of the questionnaire completed by parents or adult study participants. The following explanatory variables were recorded: maternal education (higher, secondary, primary or vocational); household monthly income per capita in Polish currency (PLN): less than 500 PLN, 501–1000 PLN, more than 1000 PLN; the area of the school location (urban big city, urban other, rural); level of schooling (primary, middle, secondary) and participation in physical education (the variable was dichotomised according to the answer to question “Is your child participating in physical education classes?”). Maternal education level and household monthly income per capita were considered as indicators of socioeconomic status (SES).

In order to examine the relationship between the level of the GDP and thinness, the area of the country was divided into regions: region 1 in which the GDP per inhabitant was above 150% of the national average, region 3 in which the GDP per inhabitant was below 80% of the national average and region 2 in which the GDP per inhabitant was between 150 and 80% of the national average [8].

Urban and rural area was defined according to the Central Statistical Office of Poland (CSO) classification. In urban areas, cities with over 500,000 residents were selected as “big cities”. Only primary and middle schools were included in the analysis of the school location, because almost all secondary schools were located in urban areas.

### Statistical analysis

Statistical analysis was performed using SAS statistical software version 9.4. The survey data were weighted against the CSO 2008 census demographic estimates. The weight construction included gender, age and region, so that the sample reflects structure of Polish population aged 7–18 years. Descriptive statistics were performed with the use of Proc Surveymeans and Proc Surveyfreq providing unweighted sample sizes and weighted means or proportions. Standard errors were adjusted for clustering at the PSU level. Thinness and overweight including obesity proportions and proportions of thinness according to the birth weight category, GDP, and schooling level categories were compared with the use of Rao-Scott Chi-Square test. The associations of thinness and birth weight categories with height expressed as z-scores relative to the Polish 2010 growth references [13] were examined with the use of multiple linear regression. Logistic regression, which incorporates a complex survey sample design, was performed to obtain adjusted odds ratios (ORs) and 95% confidence intervals (95% CIs), measuring the association between gender and other exposure variables and the odds of being thin, in univariate models. Finally, a multivariable logistic regression analysis was performed with thinness as the dependent variable. The associations were examined separately for boys and girls. Weighted percentages of non-participation in physical education classes and its ORs with 95% CIs were calculated according to the weight status categories. Values of *p* < 0.05 were considered statistically significant. A sequential Bonferroni correction was applied to adjust the significance level of multiple comparisons.

## Results

Among the 17,573 students who participated in the study, 123 were younger than 7 or older than 18 years, thus the data were not included in the analysis. In the case of 16 students, height and/or weight measurements were missing; we also excluded the data of young women who at the time of the study were pregnant (7 women). Finally, the data from 17,427 students (52.2% girls) were analysed. For some analyses, the number of participants was smaller due to missing data: 459 for maternal education, 5344 for income per capita, 1835 for birth weight and/or 226 for physical activity. Descriptive statistics of the sample: unweighted sample sizes and weighted means or proportions are given in the Table 1.

**Table 1:** Summary of study sample characteristics

Variable		Boys			Girls		
		*N* ^a^	Mean or proportion^b^	SE	*N* ^a^	Mean or proportion^b^	SE
	Age (years)	8327	13.0	0.18	9100	13.0	0.18
	Height (cm)	8327	157.8	0.97	9100	152.6	0.68
	Weight (kg)	8327	50.3	0.84	9100	45.7	0.60
	BMI (kg/m^2^)	8327	19.4	0.11	9100	19.1	0.10
	Height z-score	8327	0.0	0.01	9100	0.0	0.01
	BMI z-score	8327	0.0	0.01	9100	0.0	0.01
	Birth weight (g)	7387	3433.0	7.41	8206	3281.0	6.66
Low birth weight (<2500 g)	Yes	358	4.7	0.27	554	6.7	0.27
	No	7029	95.3	0.27	7652	93.3	0.27
Body weight category	Normal weight	5932	71.5	0.57	6550	72.1	0.55
	Overweight incl. obesity	1562	18.6	0.53	1304	14.3	0.46
	Thinness all grades	833	9.9	0.36	1246	13.6	0.42
	Thinness grade 1	713	8.4	0.34	996	10.8	0.37
	Thinness grade 2	91	1.1	0.11	193	2.1	0.17
	Thinness grade 3	29	0.4	0.07	57	0.6	0.09
Maternal education	University	1577	19.1	0.94	1611	18.1	0.92
	Secondary	3247	40.1	0.64	3431	38.4	0.72
	Primary or vocational	3280	40.7	1.12	3822	43.6	1.17
Type of school	Primary	4372	50.1	2.99	4337	49.7	2.97
	Middle	2114	27.0	2.68	2258	26.6	2.65
	Secondary	1841	22.8	2.32	2505	23.7	2.36
School location^c^	Big city	591	8.9	1.94	636	9.5	2.06
	Town or small city	3309	51.4	3.29	3353	50.5	3.29
	Rural	2586	39.7	3.10	2606	40.0	3.11
Income per capita	≤500 PLN	2819	48.4	1.14	3095	49.4	1.14
	501–1000 PLN	1850	31.9	0.72	1968	31.5	0.69
	>1000 PLN	1139	19.7	0.91	1212	19.1	0.87
Gross Domestic Product	Region 1	1124	13.0	1.03	1276	13.0	0.92
	Region 2	5253	64.0	1.51	5642	64.0	1.45
	Region 3	1950	23.1	1.33	2182	23.1	1.29
Participation in physical education	Yes	7935	96.8	0.23	8617	95.8	0.32
	No	249	3.2	0.23	400	4.2	0.32

Thinness grade 1, 2 and 3 corresponds to the BMI 17 to <18.5, 16 to <17 and <16 kg/m^2^ at the age of 18 years, respectively.

*SE* standard error, *PLN* Polish zloty

^a^Unweighted

^b^Weighted

^c^Only pupils from primary and middle schools

### Body weight distribution by BMI category and thinness grade

Thinness was less frequent than overweight including obesity (11.7 vs 16.5%; *p* < 0.01). Analysis by gender revealed that among boys, the prevalence of thinness was significantly lower compared to the prevalence of overweight including obesity (*p* < 0.01); in the group of girls, the prevalence of thinness and overweight including obesity were similar (*p* = 0.27) (Table 1).

Thinness grade 1 accounted for 9.6%, grade 2 for 1.6% and grade 3 for 0.5% of all children. Thinness grade distribution has been presented in Table 1. Female sex significantly increased odds of being thin: OR = 1.43 (CI 1.29–1.59) and OR = 1.32 (CI 1.18–1.48), OR = 1.92 (CI 1.46–2.53) and OR = 1.79 (CI 1.12–2.86) for thinness of all grades and grades 1, 2 and 3, respectively (*p* < 0.01 for thinness all grades and grades 1 and 2 and *p* = 0.016 for grade 3).

### Thinness, birth weight and body height

**Fig. 1:**
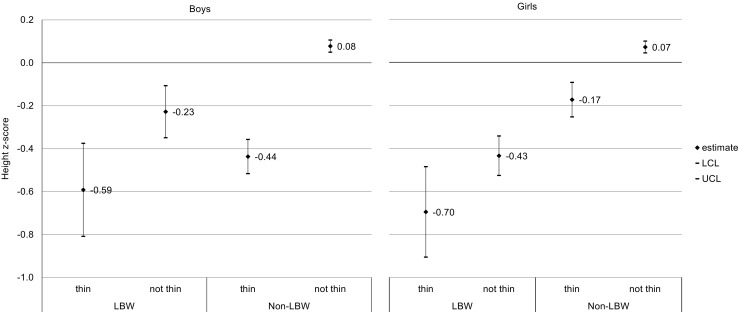
The weighted means of height z-score in thin and not thin girls and boys according to the birth weight category. *LCL*, *UCL* lower and upper confidence limit of 95% CI, *LBW* low birth weight (<2500 g). Non-LBW: birth weight ≥ 2500 g

Thinness was more prevalent in children who had low birth weight (LBW; less than 2500 g): 16.1 vs 11.6%, LBW vs non-LBW, respectively; *p* < 0.01. LBW increased odds of being thin in the case of boys (*p* < 0.01), but not in the case of girls (Table 2). The weighted mean height z-score was significantly lower in the case of thin boys and girls in comparison with not thin peers (non-overlapping confidence intervals of the mean) with the exception of LBW girls (Fig. 1.)

**Table 2:** Univariate associations of exposure variables with thinness weighted percentages

Exposure variable		Boys		Girls	
		Thinness (%)	OR (95% CI)	Thinness (%)	OR (95% CI)
Birth weight	> = 2500 g	9.8	ref.	13.6	ref.
	<2500 g	17.6	1.96 (1.44–2.66)	15.7	1.14 (0.88–1.47)
Maternal education	University	9.4	ref.	12.3	ref.
	Secondary	9.4	1.03 (0.82–1.29)	13.5	1.09 (0.90–1.32)
	Primary or vocational	10.7	1.15 (0.91–1.44)	14.3	1.14 (0.93–1.38)
Income per capita	≤500 PLN	10.9	ref.	14.3	ref.
	501–1000 PLN	9.6	0.91 (0.74–1.12)	13.8	0.98 (0.82–1.16)
	>1000 PLN	9.2	0.85 (0.65–1.10)	12.7	0.87 (0.71–1.08)
Schooling level	Primary	11.0	ref.	12.8	ref.
	Middle	10.3	0.93 (0.78–1.11)	13.7	1.09 (0.91–1.30)
	Secondary	7.0	0.61 (0.49–0.77)	15.0	1.20 (1.03–1.41)
School location^a^	Big city	10.9	ref.	10.3	ref.
	Town or small city	10.9	1.01 (0.74–1.37)	13.5	1.36 (1.02–1.81)
	Rural	10.5	0.96 (0.70–1.32)	13.3	1.34 (1.00–1.79)
Gross domestic product	Region 1	7.7	ref.	10.2	ref.
	Region 2	9.9	1.33 (0.98–1.79)	13.7	1.39 (1.13–1.71)
	Region 3	10.9	1.47 (1.06–2.05)	15.2	1.57 (1.23–1.99)

*CI* confidence interval, *OR* odds ratio, *PLN* Polish zloty

^a^Only pupils from primary and middle schools

Thinness and LBW were independently, significantly negatively associated with body height in boys and girls (Table 3).

**Table 3:** Results of multiple regression models of thinness and low birth weight as explanatory variables for body height (expressed as a z-score)

	Boys			Girls		
	Estimate^a^	SE	*p*	Estimate^a^	SE	*p*
Intercept	0.08	0.01	<0.01	0.07	0.01	<0.01
Thinness	−0.50	0.04	<0.01	−0.25	0.04	<0.01
Low birth weight	−0.28	0.06	<0.01	−0.51	0.04	<0.01

^a^Weighted

### Thinness according to maternal education and household monthly income per capita

The prevalence of thinness was highest among boys and girls of mothers whose educational level was low and in families which declared low income per capita; however, these differences lacked statistical significance: thinness weighted ORs 95% CI include 1 (Table 2).

### Thinness and GDP per inhabitant

The prevalence of thinness among students increased with a decrease in the GDP per inhabitant in regions (8.9 vs. 11.8 vs. 13.0%; *p* < 0.001). Region with the lowest GDP per inhabitant significantly increased weighted ORs of thinness (Table 2).

### Thinness according to the level of schooling and location of school

**Fig. 2:**
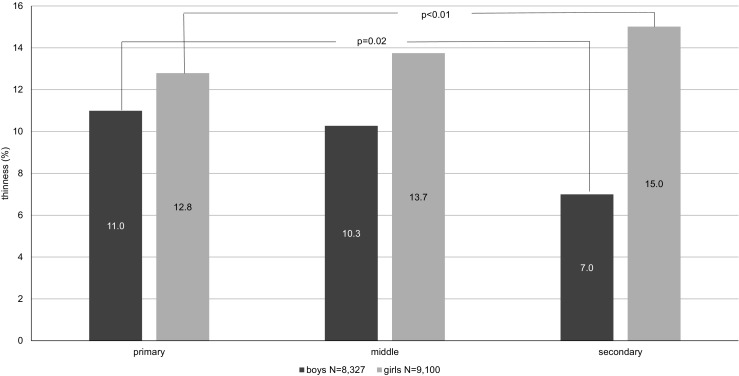
The weighted prevalence of thinness by the level of schooling

The percentage of thin students varied according to the level of schooling and therefore depended on the age of the students (primary 7–12 years; middle 13–15 years; secondary 16–18 years), whereas the direction of change depended on gender. Among boys, the weighted percentage of thinness was highest in primary schools and decreased at subsequent levels of education, while among girls, the opposite: the highest percentage of thinness was found in secondary schools and the lowest in primary schools; the differences between primary and secondary level of schooling were statistically significant (Fig. 2). In comparison to primary level, secondary level of schooling increased OR of thinness in the case of girls, but in the case of boys, it decreased (Table 2).

The highest percentage of thin girls was recorded in schools located in urban areas outside the cities inhabited by more than 500,000 residents. In girls, thinness OR was significantly higher in comparison to big city vs town or small city and vs rural. There was no significant difference in the case of boys (Table 2).

### Multivariable models for thinness

In the multiple logistic models, explanatory variables, which showed statistical significance in univariate analysis with thinness, were included with the exception of “School location”, which was not included due to the fact that school location on secondary level did not allow differentiating records to urban-rural levels. The LBW and lowest GDP region were found to be independent risk factors for thinness in the case of boys, whereas secondary level of schooling decreased odds of thinness. In the case of girls, secondary level of schooling and regions with lower than highest GDP were increasing odds of thinness (Table 4).

**Table 4:** Results of multivariable logistic regression models with thinness as dependent variable

	Boys	Girls
	Thinness OR^a^ (95% CI)	Thinness OR^a^ (95% CI)
Low birth weight	1.89 (1.38–2.57)	1.13 (0.87–1.46)
Schooling level middle vs primary	0.97 (0.81–1.16)	1.16 (0.97–1.38)
Schooling level secondary vs primary	0.63 (0.50–0.81)	1.19 (1.01–1.40)
GDP region 2 vs 1	1.27 (0.95–1.71)	1.40 (1.12–1.74)
GDP region 3 vs 1	1.39 (1.00–1.92)	1.56 (1.21–2.01)

^a^Weighted

### Participation in physical education

In both boys and girls, weighted frequency of non-participation in physical education classes was the lowest in the group of normal weight. Among girls, thinness (but not overweight), compared to normal weight, increased weighted odds of non-participation in physical education classes. There was an opposite pattern in the case of boys: weighted odds of non-participation in physical education were increased with overweight but not thinness (Table 5).

**Table 5:** Weighted percentages of students not participating in physical education classes according to the weight status groups and logistic regression analyses of the association of weight status with non-participation in physical education classes

Weight status	Boys		Girls	
	non-participation in PE classes (%)^a^	OR^a^ (95% CI)	Non-participation in PE classes (%)^a^	OR^a^ (95% CI)
Thinness	3.1	1.05 (0.69–1.61)	6.0	1.61 (1.22–2.12)
Normal weight	2.9	ref.	3.8	ref.
Overweight^b^	4.1	1.4 (1.01–1.94)	4.1	1.06 (0.77–1.47)

*PE* physical education

^a^Weighted

^b^Including obesity

## Discussion

Due to the growing epidemic of overweight and obesity, the focus of researchers remained mainly to excessive body weight [2, 7, 20, 21, 24]. Our analysis showed that although weight deficit among students was less frequent than overweight and obesity (jointly), in the group of girls, the proportion of thinness was similar to the percentage of overweight and obesity. Weight deficiency more often affected girls than boys, while overweight and obesity was more common among boys [9].

Our data show that among socioeconomic determinants, only GDP per inhabitant in the region was found to be an independent risk factor for thinness.

It should be noted that in the year preceding the survey in five Polish districts, called in paper *region 3*, GDP per inhabitant was below 40% for the European Union and only in Masovian district (*region 1*) exceeded 75% for the EU [8]. In Poland according to the CSO, the average monthly household per capita income in 2008 was 1046 PLN, i.e. about 250 Euro, and a household per capita income below 500 PLN for many years entitles the family to apply for child benefits. Generally low level of household income per capita in Poland may be related to low differentiation of thinness in relation to the economic status. In addition, lack of statistically significant association with income may be linked to relatively high frequency of missing data on income provided by the study questionnaire.

Results of our analysis with regard to low impact of material factors on thinness prevalence among children and adolescents is in line with observations of other researchers [6].When considering the shaping of body weight, gender appears to be an important factor, not only because of the scale of the phenomenon but also in the context of socio-economic, environmental and psychological determinants. Although the percentage of thinness was higher in the LBW children, only in boys, the difference was statistically significant. Similarly, differences in thinness prevalence according to age groups, from primary to secondary schooling level, show opposite trend: decrease in boys and increase in girls. It may indicate a gendered mechanism of formation and maintenance of a body weight deficiency [6].

Confirmation of the different approaches of boys and girls to their body weight can be found in the results of the study of youth aged 16–18 years, carried out in 2005 in Warsaw [17]. In this study, girls significantly more often declared that they were currently (at the time of the study) taking action to reduce body weight (26%) than were boys (9%) (*p* < 0.001); girls prevailed also among those who in the past 12 months preceding the study, in order to reduce weight, applied methods risky to health, e.g. a starvation diet, induced vomiting, use of diet pills or laxatives (15% of boys vs 25% girls, *p* < 0.001). Taking action to reduce weight was related to the subjective assessment of body weight. In all categories of body weight, statistically significant differences were found between boys and girls in the self-assessment of body weight. Boys more often assessed themselves as too thin and girls as too fat. Almost 40% of boys with excessive body weight evaluated their body mass as appropriate, and over 50% of girls with an average body weight saw themselves as too fat; 17% of underweight girls also assessed themselves as too fat [17].

It should be noted that the study of Whitaker et al. [23] indicates that paediatric thinness is associated with the familial tendency to have low body weight, and thus intergenerational thinness transmission, which supports the view that many cases of underweight represents a low level of distribution of healthy body weight having genetic origin.

In European countries, as well as in Australia, the prevalence of underweight among children and adolescents is about 4–8% [5, 14, 16]. In the Polish study, in the analysis of changes in the prevalence of underweight among children and adolescents aged 7–18 years in Łódź within the 26 years of transformation (both economic and political), Żądzińskia et al. [25] observed an increase in body weight deficiency in the years 1977/1978 and 2002/2004, in the group of boys from 7.2 to 12.1% and in the group of girls from 11.0 to 20.2%. In comparison to other European countries, it is a disturbing trend.

Researchers from the Czech Republic noted in 2001 as compared to 1991 a decrease in the percentage of students with a very low BMI among younger school-age children and a sharp increase in adolescence. In assessing this phenomenon as negatively as the increase in the percentage of obese people in the population, they discerned its causes in poor nutrition due to diets aiming to achieve a very slim figure, which is currently in fashion, and increasing anorexia, especially among teenage girls [12]. In Polish studies of secondary and high school students, the percentage of respondents who declared anorexia was 6.9%, including 4.3% of respondents with normal body weight and 1.6% with underweight. In this study, the percentage of underweight adolescents and young adults was 12.7% for males and 21.5% for females. The highest frequency of underweight was reported in rural areas, and the lowest in cities with populations of more than 100,000 residents [10].

In the current analysis, a significant difference in the prevalence of thinness in the group of school-aged girls in urban areas was reported; the lowest percentage of thinness was observed among girls in cities with populations of more than 500,000 residents. Among girls, the prevalence of thinness in the other urban areas and in rural areas was comparable. The lowest prevalence of thinness among students in big cities may be associated with the higher level of women’s education and higher income of residents of big cities (data not shown); in Poland, there are no big cities (with populations of more than 500,000 residents) in the region with low GDP per inhabitant (below 80% of the national average).

Low body mass may have a negative impact on the physical development of school-age children and adolescents. In the group of thin girls, the relatively high percentage of students who did not participate in physical education drew attention; it is greater than in the group with normal body weight and even greater than in the group with excess body weight, which can affect the physical ability and general body efficiency of thin girls. Researchers who studied differences in health-related fitness among Spanish adolescents classed as underweight, normal weight, overweight or obese according to BMI concluded that not only overweight and obesity but also underweight seem to be determinants of health-related fitness in adolescents [1].

The average body height of thin girls and boys, independent of birth weight, was significantly lower than of students who were not thin. It may indicate that underweight children do not realize their full potential in terms of body height. However, due to the fact that the study did not examine the relationship between BMI of children and parents, the effect of the primary limitations of biological and/or cultural potential for the occurrence of underweight cannot be excluded.

As reported by other researchers, in the case of girls, underweight is of particular importance in the context of future motherhood [11, 19]. The number of infants born before 32 weeks was significantly higher in the group of underweight mothers, compared to the group with normal weight before pregnancy. Underweight mothers were at increased risk for low birth weight and very low birth weight of newborns. In addition, deficient female body weight before pregnancy may pose a risk to the newborn of developing respiratory distress syndrome and anaemia [11, 19]. In this context, the increase in the proportion of girls with a deficiency of body weight is a phenomenon of a negative nature, the consequences of which can be transferred to the next generation.

## Strength and limitations

The main strength of this study is that it is a large, nationally representative sample of school-aged children and adolescents which provided data on socioeconomic status and measured height and weight.

Statistical analysis accounted for complex sampling design of the survey; all analyses applied weights and cluster structure of the two-stage sample.

The main limitation of the study is response rate of 71% and the relatively high frequency of missing income data.

## Conclusions

For the first time, the prevalence and determinants of thinness based on data from a nationally representative, weighted sample of Polish children aged 7–18 years were presented. Gender and the socioeconomic factors, GDP region, were important determinants of thinness among Polish children and adolescents. The analysis shows how socioeconomics determinants and gender influence the prevalence of thinness among children and adolescents in post-transformation country and can be used for international comparisons. Public health strategies should address family eating practices to prevent negative effects of weight deficit, especially among girls/children from low GDP regions.

## Abbreviations

BMI: Body mass index
CI: Confidence interval
CMHI: Children’s Memorial Health Institute
CSO: Central Statistical Office
EU: European Union
GDP: Gross domestic product
IOTF: International Obesity Task Force
LBW: Low birth weight
OLAF: The “Elaboration of reference blood pressure ranges for children and adolescents in Poland” PL0080 study
OR: Odds ratio
PE: Physical education
PLN: Polish zloty (units of Polish currency)
PSU: Primary sampling unit
SE: Standard error
SES: Socioeconomic status

## References

[1] Artero EG, España-Romero V, Ortega FB, Jiménez-Pavón D, Ruiz JR, Vicente-Rodríguez G, Bueno M, Marcos A, Gómez-Martínez S, Urzanqui A, González-Gross M, Moreno LA, Gutiérrez A, Castillo MJ (2010). Health-related fitness in adolescents: underweight, and not only overweight, as an influencing factor. The AVENA study. Scand J Med Sci Sports.

[2] Bac A, Woźniacka R, Matusik S, Golec J, Golec E (2012). Prevalence of overweight and obesity in children aged 6–13 years—alarming increase in obesity in Cracow, Poland. Eur J Pediatr.

[3] Cole TJ, Bellizzi MC, Flegal KM, Dietz WH (2000). Establishing a standard definition for child overweight and obesity worldwide: international survey. Brit Med J.

[4] Cole TJ, Flegal KM, Nicholls D, Jackson AA (2007). Body mass index cut offs to define thinness in children and adolescents: international survey. Brit Med J.

[5] Ferrar K, Olds T (2010). Thin adolescents: who are they? What do they do? Socio-demographic and use-of-time characteristics. Prev Med.

[6] Godley J, McLaren L (2010). Socioeconomic status and body mass index in Canada: exploring measures and mechanisms. Can Rev Sociol.

[7] Goldfield GS, Murray M, Maras D, Wilson AL, Phillips P, Kenny GP, Hadjiyannakis S, Alberga A, Cameron JD, Tulluch H, Sigal RJ (2016). Screen time is associated with depressive symptomatology among obese adolescents: a HEARTY study. Eur J Pediatr.

[8] Gross Domestic Product. Regional accounts in 2007. Central Statistical Office, Statistical Office in Katowice. Katowice 2009. http://stat.gov.pl/cps/rde/xbcr/gus/rn_pkb_rachunki_regionalne_w_2007.pdf. Accessed 30 May 2016

[9] Gurzkowska B, Kułaga Z, Litwin M, Grajda A, Świąder A, Kułaga K, Góźdź M, Wojtyło M (2014). The relationship between selected socioeconomic factors and basic anthropometric parameters of school-aged children and adolescents in Poland. Eur J Pediatr.

[10] Kapka-Skrzypczak L, Bergier B, Diatczyk J, Niedźwiecka J, Biliński P, Wojtyła A (2012). Dietary habits and body image perception among polish adolescents and young adults—a population based study. Ann Agric Environ Med.

[11] Kaźmierczak J, Reszczyńska M, Szymański W, Daniłko M (2009). Znaczenie masy ciała matek dla przebiegu porodu oraz adaptacji okołoporodowej noworodków. Perinatol Neonatol Ginekol.

[12] Kobzová J, Vignerová J, Bláha P, Krejčovsky L, Riedlová J (2004). The 6^th^ nationwide anthropological survey of children and adolescents in the Czech Republic in 2001. Cent Eur J Public Health.

[13] Kułaga Z, Litwin M, Tkaczyk M, Palczewska I, Zajączkowska M, Zwolińska D, Krynicki T, Wasilewska A, Moczulska A, Morawiec-Knysak A, Barwicka K, Grajda A, Gurzkowska B, Napieralska E, Pan H (2011). Polish 2010 growth references for school-aged children and adolescents. Eur J Pediatr.

[14] Lazzeri G, Ross S, Pammolli A, Pilato V, Pozzi T, Giacchi MV (2008). Underweight and overweight among children and adolescents in Tuscany (Italy). Prevalence and short-term trends J Prev Med Hyg.

[15] Lusky A, Barell V, Lubin F, Kaplan G, Layani V, Shohat Z, Lev B, Wiener M (1996). Relationship between morbidity and extreme values of body mass index in adolescents. Int J Epidemiol.

[16] Marques-Vidal P, Ferreira R, Oliviera JM, Paccaud F (2008). Is thinness more prevalent than obesity in Portuguese adolescents?. Clin Nutr.

[17] Oblacińska A, Tabak I, Jodkowska M (2007). Eating, dieting and other weight control behaviors of Polish adolescents 16-18- years in the context of body image and body weight perception. Prob Hig Epidemiol.

[18] Pietrobelli A, Faith MS, Allison DB, Gallagher D, Chiumello G, Heymsfield S (1998). Body mass index as a measure of adiposity among children and adolescents: a validation study. J Pediatr.

[19] Salihu HM, Lynch O, Alio AP, Mbah AK, Kornosky JL, Marty PJ (2009). Extreme maternal underweight and feto-infant morbidity outcomes: a population-based study. J Maternal-Fetal Neo M.

[20] Sanders RH, Han A, Baker JS, Cobley S (2015). Childhood obesity and its physical and psychological co-morbidities: a systematic review of Australian children and adolescents. Eur J Pediatr.

[21] Sousa P, Fonseca H, Gaspar P, Gaspar F (2015). Controlled trial of an Internet-based intervention for overweight teens: effectiveness analysis. Eur J Pediatr.

[22] Suliga E (2006). Anthropometrical methods of assessing nutritional status of children and adolescents. Pediatr Pol.

[23] Whitaker KL, Jarvis MJ, Wardle J (2011). The intergenerational transmission of thinness. Arch Pediat Adol Med.

[24] Wijnhoven T, van Raaij J, Spinelli A (2014). WHO European childhood obesity surveillance initiative: body mass index and level of overweight among 6–9-year-old children from school year 2007/2008 to school year 2009/2010. BMC Public Health.

[25] Żądzińska E, Rosset I, Kozieł S, Nawarycz T, Borowska-Strugińska B, Lorkiewicz W, Ostrowska-Nawarycz L, Sitek A (2012). Frequency of under- and overweight among children and adolescents during the economic transition in Poland. Homo.

